# Current advances of CRISPR-Cas technology in cell therapy

**DOI:** 10.1016/j.cellin.2022.100067

**Published:** 2022-10-26

**Authors:** Hou-Yuan Qiu, Rui-Jin Ji, Ying Zhang

**Affiliations:** Department of Rheumatology and Immunology, Medical Research Institute, Frontier Science Center for Immunology and Metabolism, Zhongnan Hospital of Wuhan University, Wuhan University, Wuhan, 430071, China

**Keywords:** CRISPR, Cas9, Cell therapy, Genome editing, HSPC, T cell, iPSC

## Abstract

CRISPR-Cas is a versatile genome editing technology that has been broadly applied in both basic research and translation medicine. Ever since its discovery, the bacterial derived endonucleases have been engineered to a collection of robust genome-editing tools for introducing frameshift mutations or base conversions at site-specific loci. Since the initiation of first-in-human trial in 2016, CRISPR-Cas has been tested in 57 cell therapy trials, 38 of which focusing on engineered CAR-T cells and TCR-T cells for cancer malignancies, 15 trials of engineered hematopoietic stem cells treating hemoglobinopathies, leukemia and AIDS, and 4 trials of engineered iPSCs for diabetes and cancer. Here, we aim to review the recent breakthroughs of CRISPR technology and highlight their applications in cell therapy.

## 1 Introduction

The discovery of CRISPR-Cas nucleases has revolutionarily transformed both basic research and emerging therapeutic modalities. CRISPR-Cas system, such as *Streptococcus pyogenes* Cas9 (SpCas9), are composed of Cas9 endonuclease and a single guide RNA (sgRNA), in which Cas9 cleaves DNA and sgRNA provides target specificity through base-pairing with DNA (Jinek et al., 2012). In addition to sgRNA directed base-pairing, a protospacer adjacent motif (PAM) sequence located in the target sequence is essential in initiating Cas9 recognition and binding, thereby conferring the subsequent base-pairing with sgRNA and cleavage. After cleavage, a double strand break (DSB) is created and cells initiate DNA damage repair pathways to reseal the break. The ability of Cas9 to precisely edit genome with high efficiency and the ease of engineering sgRNA sequence to direct any site as long as a PAM sequence is present, have led to the development of many biological tools. Cell therapy is an emerging therapeutic modality, which involves the genetic manipulation of cells outside of body and infusing edited cells back into patients to provide therapeutic benefits. In this review, we describe the development of different CRISPR-Cas based tools and how these tools have impacted cell therapy. Finally, we discuss the challenges when applying in the current clinical trials and highlight future directions that enlighten translational research.

## 2 CRISPR toolbox for translational study

CRISPR-Cas system, originally identified as prokaryotic adaptive immune system to protest host from phage invasions, existed in 85.2% of archaea and 42.3% of bacteria (Makarova et al., 2020). Thanks to the advance of metagenomic sequencing, many CRISPR nucleases have been identified and more than a hundred nucleases have been characterized. Among them, three nucleases (SpCas9, AsCas12a, SaCas9) are being tested in clinical studies due to their superior characteristics such as high editing efficiency, simple PAM sequence or small size that enables viral package for delivery. Here, we will review the basics of CRISPR toolbox in the translational study and the advances that have been made upon these Cas nucleases.

### 2.1 1st generation: The fundamental CRISPR-Cas

**Fig. 1:**
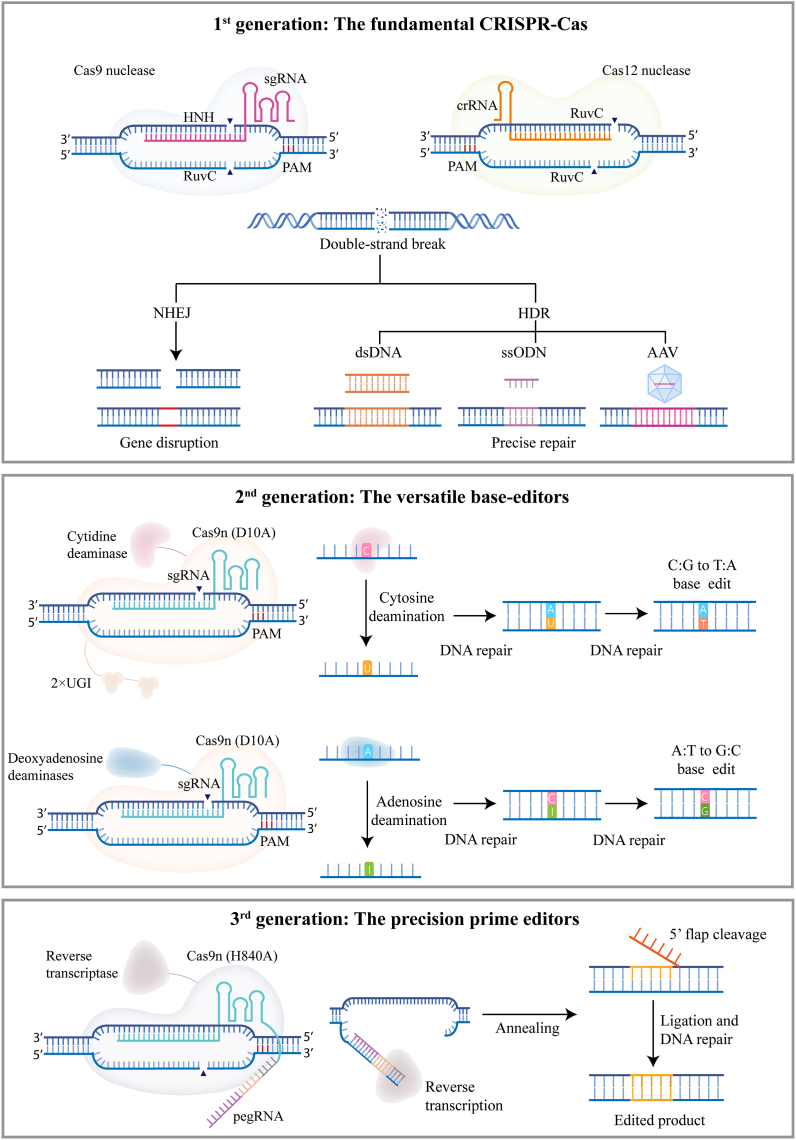
| **CRISPR toolbox for translational study.** Illustration of three generations of CRISPR-based genome editing tools: nucleases, base editors and prime editors. Examples of substrates and edited products are shown, along with CRISPR-Cas tools that can be used to achieve the target editing. PAM, protospacer adjacent motif; NHEJ, non-homologous end joining; HDR, homology-directed repair; UGI, uracil glycosylase inhibitor (UGI).

Cas9 nucleases, particular *Streptococcus pyogenes* Cas9 (herein SpCas9), has the highest mammalian cell editing activity of all CRISPR nucleases discovered. Cas9 has two nuclease domains, HNH and RuvC, each responsible to cleave the target and non-target strand of DNA, respectively (Fig. 1) (Gasiunas et al., 2012; Jinek et al., 2012). When guided by sgRNA, SpCas9 generates DSBs at the target locus, which activates cell repair machineries to reseal the DSBs (Lieber et al., 2003; Price and D'Andrea, 2013). Non-homologous end joining (NHEJ) and homology directed recombination (HDR) are the two major repair pathways. NHEJ tends to introduce small insertions and deletions (Indels) around the break, thereby resulting gene disruption and decreased protein expression. In contrast to NHEJ mediated gene disruption, when presented with donor DNA template, HDR pathway could perfectly repair the mutation or insert a gene of interest into target locus (Platt et al., 2014). NHEJ functions at all stages of cells whereas HDR requires cells in the dividing stage, the latter of which makes the HDR less efficient than NHEJ (Heyer et al., 2010; Lieber, 2010; Lin et al., 2014). SpCas9 has a PAM of NGG (N = A/T/C/G), which theoretically covers 12.5% human genome. The high cleavage efficiency in mammalian cells and the flexibility of PAM selections make SpCas9 the most widely used nucleases that goes into many clinical trials. SaCas9, identified from *Staphylococcus aureus,* has a PAM of NNGRRT (R = A/G, N = A/T/C/G) (Kleinstiver et al., 2015; Ran et al., 2015). SaCas9 is 1 kb smaller than SpCas9, making it possible to be packaged into single adeno-associated virus (AAV) vector for delivery and is now harnessed for treating leber congenital amaurosis type 10 (Maeder et al., 2019; Ran et al., 2015). Unlike SpCas9 or SaCas9, AsCas12a derived from *Acidaminococcus* sp., only carries one nuclease domain RuvC that functions to cleave both target and non-target strand (Fig. 1) (Jeon et al., 2018; Shmakov et al., 2015; Zetsche et al., 2015). AsCas12a has a PAM of TTTV (V = A/C/G) and the cleavage site is far from PAM sequence, making it unique in editing thymine-rich regions (Kim et al., 2017). The original AsCas12a exhibits relative low activity. To improve its efficiency, Zhang et al. performed directed evolution in bacterial and isolated a variant carrying M537R/F870L double mutations, referred as AsCas12a ULTRA (Zhang et al., 2021). AsCas12a ULTRA has been shown to achieve effective multiplex editing in primary T cells and remain high activity in other therapeutic cell types. Currently, Cas12a variants are being tested in clinical trials to treat sickle cell disease (SCD) and transfusion dependent β-thalassemia (De Dreuzy et al., 2019, NCT04853576, NCT05444894).

### 2.2 2nd generation: The versatile base-editors

Most genetic disorders are caused by single mutations, and direct correction of mutated bases offers an effective path to cure. While Cas9 could correct a point mutation if presented with a repair donor, the dependence of cell cycling makes it impossible to achieve therapeutic threshold, as most therapeutic relevant cells are non-cycling or slow cycling (Lin et al., 2014; Paquet et al., 2016). In order to overcome the dependence of HDR repair pathway, researchers developed base editors by fusing a cytosine deaminase or engineered adenine deaminase to a catalytically impaired Cas9 nuclease (D10A). Two main classes of base editors have been developed: cytosine base editor (CBE) or adenine base editor (ABE), which converts C-to-T, G-to-A or A-to-G, T-to-C, respectively (Fig. 1) (Gaudelli et al., 2017; Komor et al., 2016). In cases of CBE, cytosine deaminase catalyzes the removal of amine group in cytosines to generate uridines in the non-target strand, resulting a U:G mismatch that can be repaired to U:A, and further read as T:A by polymerase. To prevent the cleavage of U in the U:G mismatch, a uracil glycosylase inhibitor (UGI) is added to CBE which substantially enhances the editing efficiency (Anzalone et al., 2020). In cases of ABE, since there is no natural deoxyadenine deaminase, researchers performed a thorough protein evolution based on an RNA-specific adenine deaminase TadA in *Escherichia coli* (Gaudelli et al., 2017). After seven rounds of evolution, the engineered TadA, also referred as ABE7.10 is able to catalyze the removal of amine group of deoxyadenine to inosinces, the latter of which is recognized as guanines during repair. In addition to CBE and ABE, new base editors that mediate C-to-G conversion or simultaneously converting C-to-T and A-to-G have also been developed (Grunewald et al., 2020; Li et al., 2020; Sakata et al., 2020; Zhang et al., 2020b).

Although base editors represent a powerful tool to precisely restore certain types of point mutations without the need of DNA repair templates or generating DSBs, and the process is cell-cycle independent, there are still several limitations such as bystander effects whereby unwanted cytosines or adenines are converted and the severe off-target effects of CBE (Gaudelli et al., 2017; Komor et al., 2016, 2017; Nishida et al., 2016). Many efforts have been devoted in engineering a better variant that could meet the demands. Since a detailed summary of each BE variant have been described in other reviews (Porto et al., 2020; Rees and Liu, 2018), we choose to focus on the variants that have been tested in preclinical or clinical studies. BE4max, composed of codon-optimized rAPOBEC1 deaminase, two copies of UGIs, and optimized nuclear localization signal (NLS), is one of the most advanced and potent CBE (Koblan et al., 2018). BE4max has an editing window of position 3–9 counting from protospacer and a sequence preference of TC, preferentially editing cytosine when a thymine was nearby (Koblan et al., 2018). The sequence preference helps decrease the bystander editing and enrich the desired editing. Due to the high activity of cytosine deaminase, many CBEs including BE4max have been shown to cause sgRNA independent off-targets (Wang et al., 2021; Zhang et al., 2020a). A3A (N57Q)-BE3 is another variant that was engineered to show significantly reduced off-targets (Gehrke et al., 2018; Zeng et al., 2020). Transformer base editor (tBE) is an example of mitigating off-target activity of CBE by introducing a control-release deaminase inhibitor into the system (Wang et al., 2021). Different than CBE, ABE has relative low deaminase activity as evidenced by *in vitro* deaminase assay (Lapinaite et al., 2020). Most efforts have been focused on improving its activity either by directed evolution or saturated mutational screen. Among them, ABE8e or ABE8.20 have been developed by different groups to show up to 1000-fold enhancement of deamination activity than the original version ABE7.10 (Gaudelli et al., 2020; Lapinaite et al., 2020; Richter et al., 2020). In case of direct restoration of pathogenic mutation, the restriction of editing window and the requirement of a PAM sequence together make it difficult to identify a sgRNA around the pathogenic site. To solve the issue, several efforts have been made to engineer a PAM-less Cas9 variants (Collias and Beisel, 2021; Walton et al., 2020). Through phage-assisted non-continuous evolution, ABE8e-NRCH was developed, which has a PAM preference of NG (N = A/T/C/G) (Miller et al., 2020) and exhibited high editing efficiency to repair a sickle cell mutation in hematopoietic stem cells (Newby et al., 2021). A clinical trial is also initiated to treat sickle cell disease based on the ABE (NCT05456880).

### 2.3 3rd generation: The precision prime editors

The discovery of the fundamental Cas9 genome editing tools has enabled efficient gene disruption. Base editors further opened the opportunity to correct certain pathogenic mutations. However, the limitation of certain base conversions (C-to-T, G-to-A or A-to-G, T-to-C), the generation of undesired bystander editing, and the restriction of PAM availability prevent its application to many pathogenic mutations that require precise base restorations. To fulfill the gap, prime editor was recently developed to make all 12 possible types of point mutations, small insertion and deletions in a targeted and precise manner (Anzalone et al., 2019).

Prime editor (PE) consists of nicked Cas9(H840A) fused with reverse transcriptase (RT) and a prime editing guide RNA (pegRNA) (Fig. 1) (Anzalone et al., 2019). The pegRNA is a single RNA composed of sgRNA fused with a primer binding site (PBS) and an RT template (RTT). Within the pegRNA design, the classic sgRNA structure provides sequence specificity and guides the generation of a nick in non-target strand; the PBS are designed to hybridize with the released non-target strand and RTT encoding the desire edits help guide the reverse transcription using RTT as template (Fig. 1). After reverse transcription, the RT template of pegRNA is transcribed into DNA and generates a 3’ DNA flap, which is integrated into the target site (Anzalone et al., 2019). PE has been demonstrated to successfully convert small base changes in primary mouse cortical neurons and zebrafish embryos (Anzalone et al., 2019; Petri et al., 2022). More importantly, PE mediated base conversion is cell-cycle independent (Anzalone et al., 2019). Off-target analysis of PE also suggest it is a faithful genome editing tool with no detectable pegRNA-independent off-target effects (Gao et al., 2022; Jin et al., 2021). Although PE is flexible in mediating all possible small conversions, the highly variable editing efficiency across genomic loci and cell types has limited its broad applications. In order to improve its activity, several strategies have been applied: (1) enhancing the stability of pegRNA by introducing a hairpin structure at 3′ terminus of pegRNA to avoid nuclease degradation (Nelson et al., 2022; Ying Feng et al., 2022) or formulating pegRNA as circular topology to enhance stability (Liu et al., 2022); (2) introducing synonymous mutations in the RTT of pegRNA to enhance desired editing during repair (Li et al., 2022); (3) including a second nick at 50 bp from the initial nick to enrich the desired edits (Chen et al., 2021a); (4) inhibiting mismatch repair pathway by adding a dominant negative MMR protein into PE system (Chen et al., 2021a); (5) enhancing reverse transcriptase activity by protein engineering (Zong et al., 2022); (6) enhancing nuclear delivery of PE system by optimizing nuclear localization signals (Liu et al., 2021a).

While PE allows flexible editing of few bases, large fragment insertion in non-cycling cells is still a bottle neck. To overcome this, twin PE was developed by using a pair pegRNAs plus a Bxb1 integrase into PE system (Anzalone et al., 2022). A 38-bp Bxb1 attB site and 50-bp Bxb1 attP site are inserted at specific genomic sites via PE. Bxb1 recognizes preinstalled attB and attP sites and mediates up to 5.6 kb DNA insertion if provided a donor DNA. Similarly, a different group developed GRAND editing (genome editing by RTTs partially aligned to each other but nonhomologous to target sequences within duo pegRNA) which also undertook a pair of PEs to enable insertion of 150 bp or 1 kb nucleotides with high efficiency (Wang et al., 2022). While PE based tools such as Twin PE or GRAND editing bring a highly versatile platform to precision genome editing, their relative low efficiency and delivery of prime editor protein and pegRNA or donor DNA still remain to be solved prior to broad application and therapeutic development.

## 3 Delivery gene editing tools for cell therapy

Cells used for cell therapy are all primary cells with intact innate immune system. Introducing foreign macromolecules like DNA, RNA or protein could trigger the activation of immune responses, leading to cell function disruption or cell death. It is critical to effectively delivery CRISPR system into cells and ensure a transient delivery without triggering cellular innate pathways.

***Electroporation*:** To achieve sufficient edited cells for cell replacement therapy, cells are manufactured in suspension to ensure large quantity. Electroporation is the most well-established method to delivery macromolecules such as DNA, RNA or protein into suspension cells with high efficiency. Compared to viral based methods, CRISPR system delivered via electroporation are transiently expressed without causing the risks associated with viral vector based delivery (Glass et al., 2018; Kim et al., 2014; Lino et al., 2018). Electroporation applies an electrical pulse to permeabilize cell membranes so that macromolecules can pass through the pores and diffuse into the cytoplasm (Rols, 2008). By far, electroporation has proven to be more efficient than other non-viral methods in delivering CRISPR system in suspension cells of various cell types. For example, electroporation of Cas9 ribonucleoprotein (RNP) can achieve more than 98% gene knockout efficiency in therapeutic relevant T cells and hematopoietic stem and progenitor cells (HSPCs) (Seki and Rutz, 2018; Wu et al., 2019). Electroporators come with different scales that can mediate wide ranges of cell transfection from 1E4 to 2E10 cells in a closed system, fulfilling the need for clinical scale cell manipulation. Although effective, electroporation can induce cellular toxicity if cells fail to recover from permeabilized condition and undergo cell death, a situation also known as irreversible permeabilization (Batista Napotnik et al., 2021). So careful evaluation of electroporation voltage, exposure duration and electroporation buffer are important to minimize the toxic effects on cells (Geng and Lu, 2013). Other non-viral methods such as lipid nano particle mediated mRNA delivery could circumvent the electroporation induced cellular toxicity, but its delivery efficiency requires further improvement to meet the demands (Billingsley et al., 2020).

***sgRNA or pegRNA*:** In case of SpCas9, sgRNA is a 99 nt single-stranded RNA, and the length of pegRNA depends on the PBS and RTT sequence, usually composed of 120 nt or more. sgRNA can be prepared either by *in vitro* transcription (IVT) or solid-phase synthesis. *In vitro* transcribed sgRNA carries a 5′ tri-phosphorylation and can be recognized by RIG-I to trigger cellular immune response, leading to cell toxicity and cell death (Kim et al., 2004; Mu et al., 2019; Pichlmair et al., 2006). If using IVT sgRNA, 5′ tri-phosphorylation has to be removed by alkaline phosphatase treatment (Kim et al., 2018b; Mu et al., 2019). While IVT sgRNAs are usually used for sgRNA screening due to the ease of generation, solid-phase synthesis of sgRNA with end chemical modification allows increased stability and is commonly used in clinical trials. Specifically, 2′-O-methyl 3'phosphorothioate of three terminal nucleotides at both ends significantly improved editing efficiency and thus are widely adopted for sgRNA synthesis (Hendel et al., 2015).

***Cas9 and its derivatives***: Cas9 can be formulated as purified protein or messenger RNA (mRNA) that is co-delivered with sgRNA into cells via electroporation. Cas9 protein is usually purified from *E.coli* and there are many commercial available Cas9 protein and its common variants to be purchased for direct use. Cas9-fusion proteins such as base editor and prime editor are not commercially available and can be difficult to purify due to their large size. In brief, plasmid encoding Cas9-fusion proteins was cloned and transfected into production strain. To purify protein, affinity purification and molecular sieve can be used to enrich target protein. The entire processes take a week in the lab and can be tedious. Messenger RNA is an alternative to protein, not only due to its simplicity in production but also its robust expression when transfected into cells. mRNA can be transcribed using T7 RNA polymerase and followed by the addition of 5′ capping and 3’ polyA tailing. The stability of both mRNA nuclease and its translated protein product are the critical determinants for effective genome editing. Comparison of mRNA and protein delivery has suggested that the half-live of mRNA is longer than direct protein delivery (Chen et al., 2021b; Jemielity et al., 2003; Sahin et al., 2014). Chemical modifications such as pseudouridine (Ψ), N1-methyl pseudouridine (N1-me-Ψ), 5-methyl uridine(5meU) and 5-methylcytidine (m5C) have been shown to further improve the half-lives of mRNA and suppress immune activation, resulting better genome editing outcome (Chen et al., 2021b; Kariko et al., 2008; Kormann et al., 2011; Vaidyanathan et al., 2018). Codon optimization and U depletion of mRNA sequence could also increase editing outcome likely by affecting the translation efficiency (Presnyak et al., 2015; Vaidyanathan et al., 2018). Purification by high-performance liquid chromatography (HPLC) can remove the secondary structure and maintain a homogenous pure mRNA product, which could help avoid the immunogenicity of mRNA and lead to prolonged expression (Kariko et al., 2011). Protein or mRNA formulation of Cas9 have been tested in clinical trials (Table 1). For large nucleases such as base editors and prime editors, mRNA delivery holds great potential due to its robust manufactory process and prolonged expression.

**Table 1:** | Ongoing clinical trials with CRISPR-based cell therapy.

Title	Strategy	Cell type	Trial ID
**Hemoglobinopathies**			
Transfusion-Dependent β Thalassemia	Disruption of *BCL11A* enhancer	HSPC	NCT03655678 NCT05329649 NCT05356195 NCT04925206 NCT04211480
Transfusion-Dependent β Thalassemia	Correction of CVS-654 mutation in *HBB*	HSPC	NCT04205435
Sickle Cell Disease	Disruption of *BCL11A* enhancer	HSPC	NCT03745287 NCT04443907 NCT05456880
Sickle Cell Disease	Correction of sickle mutation by HDR	HSPC	NCT04774536 NCT04819841
Sickle Cell Disease and β Thalassemia	Disruption of negative regulator binding sites at *HBG1/2* promoter	HSPC	NCT04853576 NCT05444894 NCT05456880
**Cancer malignancies**			
Leukemia	Disruption of *CD33* gene	HSPC	NCT04849910
Leukemia	CD19-CAR insertion and *PD1* knockout	T cell	NCT04213469 NCT03298828
Leukemia	BCMA-CAR T with *PD1* knockout	T cell	NCT05308875 NCT03492268
Leukemia	*CD52* and *TRAC* knockout	T cell	NCT04557436
Leukemia	Universal CAR-T	T cell	NCT04035434 NCT04637763 NCT03545815 NCT03166878 NCT03232619 NCT04264078 NCT03398967 NCT04984356 NCT05332054 NCT03229876 NCT04502446 NCT04154709 NCT04227015 NCT04026100 NCT04438083 NCT03752541 NCT04244656
Leukemia	Universal TCR-T	T cell	NCT05066165
Leukemia	Base editor generated CD7 CAR-T	T cell	NCT05397184
Leukemia	knockout *CD5*	T cell	NCT04767308
Leukemia	Disruption of *CD38* and expression of IL-15/IL-15R fusion protein	iPSC	NCT04614636
Leukemia	CD19 CAR insertion	iPSC	NCT04629729
Solid tumor	*PD-1* knockout	T cell	NCT02793856 NCT03081715 NCT03525652 NCT03044743 NCT02863913 NCT04417764
Solid tumor	*CISH* knockout	T cell	NCT04426669
Solid tumor	Mesothelin-target CAR-T and *PD1* knockout	T cell	NCT03747965
Solid tumor	anti-MUC1 CAR-T with *PD1* knockout	T cell	NCT03525782
Solid tumor	anti-prostate-specific-membrane-antigen (PSMA) CAR with *PD1* knockout	T cell	NCT04768608
Solid tumor	Engineered TILs/CAR-TILs with *PD1* Knockout and Anti-PD1/CTLA4-scFv Secreting	T cell	NCT04842812

Solid tumor	EGFR Targeted *TGFβR*-KO CAR T	T cell	NCT04976218
Solid tumor	Replacement of endogenous TCR-T with tumor specific TCR	T cell	NCT03399448
Solid tumor	Disruption of *CD38* and expression of IL-15/IL-15R fusion protein	iPSC	NCT05069935
**Infectious Disease**			
HIV-1 infection	Disruption of *CCR5* gene	HSPC	NCT03164135
**Diabetes**			
Type 1 Diabetes Mellitus	Disruption of *β2M* and overexpress PD-L1	iPSC	NCT05210530

## 4 CRISPR-based cell therapy

**Fig. 2:**
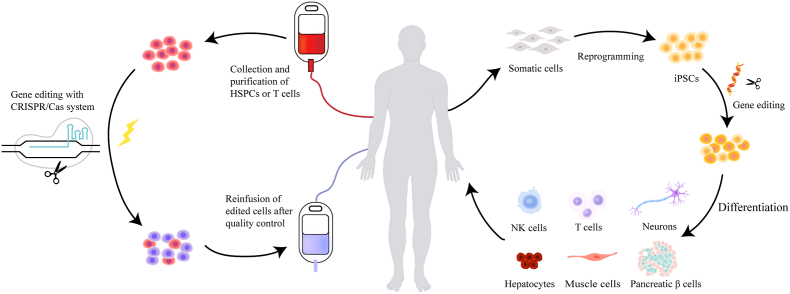
| **The flow chart of CRISPR-based cell therapy.** (**Left**) CRISPR-mediated cell therapies begin with the isolation of cells. Cells are cultured *ex vivo* and edited with CRISPR-tools before infusing back to patient. (**Right**) CRISPR-edited iPSCs therapy. iPSCs originally reprogramed from somatic cells can serve as a powerful cell source for various cell based therapies. CRISPR edited iPSCs are differentiated into cell types of interest, and differentiated cells are then transfused back into patient. iPSCs, induced pluripotent stem cells; NK cells, natural killer cells.

Cell therapy can be used to provide therapeutic benefits in multiple organs in many clinical indications. Although the specific mechanisms of each therapy vary case by case, they can be categorized into two principles by which cells achieve their therapeutic action: 1) lineage committed stem cells or progenitor cells that can repair damaged tissue. 2) cells that are able to release soluble factors such as growth factors, chemokines and cytokines, which act in a paracrine manner to target tissue or cell (Fig. 2). CRISPR-Cas system could participate in both ways either by genetically modifying the status of stem cells or progenitor cells, or by manipulating the secretion ability of cells. Here, we review how CRISPR-Cas systems accelerate the development of cell therapies in different cell types.

### 4.1 CRISPR editing in hematopoietic stem and progenitor cells (HSPCs)

HSPCs are multilineage precursor cells that are able to self-renew and differentiate to reconstitute the entire blood system, including red and white blood cells (Laurenti and Gottgens, 2018). Due to the clear isolation process of HSPCs and the ability to repopulate the blood system, hematopoietic stem cell transplantation (HSCT) has been widely applied in clinics to treat a series of blood and metabolic diseases (Chabannon et al., 2018). The major challenges associated with HSCT is to identify a matched donor and the side effects of graft versus-host disease (GvHD) using allogenic donor (Styczynski et al., 2020). In many genetic hematological disorders, mutations in HSPCs are the root of disease onset, and direct repair of these mutations using autologous HSPCs can be curative. With the discovery of robust CRISPR genome editing technology, it makes gene modification of autologous HSCT possible and opens new therapeutic opportunities to many genetic diseases that previously have no treatment. As of now, there are 15 CRISPR-edited HSPCs cell therapy going into clinical trials (Table 1).

#### 4.1.1 Hemoglobinopathies

Hemoglobinopathies, such as β-thalassemia and sickle cell disease (SCD), are the most common genetic diseases in the world. It's estimated that there are more than 40,000 newborns with β-thalassemia and 300,000 infants with SCD per year (Modell and Darlison, 2008; Piel et al., 2013). β-thalassemia and SCD are caused by different mutations in hemoglobin subunit beta (*HBB*) gene, leading to impaired β-globin production (Weatherall, 2001). Current therapies involve regular blood transfusions (often every 2–4 weeks) and iron chelation therapy, which are supportive care to relieve symptoms (Cappellini et al., 2018). Allogenic HSCT can cure the disease but identifying a matched donor and the side effects of GvHD has limited its application. Direct genetic manipulation in autologous HSPCs to restore globin expression offers a promising and curative alternative. Current strategies using CRISPR system can be divided into two categories: 1) reactivate the γ-globin gene expression to compensate the defective β-globin level and 2) direct restoration of genetic mutations in *HBB* genes particular for treating SCD. Gamma-globin was encoded by hemoglobin subunit gamma 1 (*HBG1*) gene and hemoglobin subunit gamma 2 (*HBG2*) gene, a gene duplication product with more than 97.5% sequence similarity (Wilber et al., 2011). *HBG* expression was tightly regulated during development and was silenced by regulators via binding to its promoter region (Albitar et al., 1992; Wang and Thein, 2018). To reactivate *HBG* expression, strategies such as knockout negative regulators or disrupting the binding sites on *HBG* promoter have been shown to alleviate repression and restore γ-globin expression (Frangoul et al., 2021; Newby et al., 2021; Park et al., 2019; Traxler et al., 2016; Wu et al., 2019; Zeng et al., 2020). Through genome-wide association studies, polymorphisms in the erythroid specific enhancer of gene *BCL11A* (BAF chromatin remodeling complex subunit BCL11A) were identified to have strong association with γ-globin expression (Hardison and Blobel, 2013; Sankaran et al., 2008). Disruption of these enhancer sites by SpCas9 in HSPCs resulted in erythroid-specific downregulation of *BCL11A* expression, thereby conferring elevated γ-globin induction (Fu et al., 2022; Wu et al., 2019). Using the same strategy, a Phase 2/3 clinical trial targeting *BCL11A* erythroid-specific enhancer has been initiated to treat β-thalassemia and sickle cell disease (Frangoul et al., 2021, NCT03745287, NCT03655678, NCT04208529, NCT05329649, NCT05356195). Patients treated with CRISPR-edited autologous HSPCs have become transfusion-independent and their fetal hemoglobin level sustained. This is the first peer-reviewed report showing the beneficial response after treating with CRISPR-edited HSPCs. Ever since its launch and the promising data, more trials using similar strategies have been initiated worldwide (Table 1). It's worth noting that two scientific research teams independently discovered that DSBs produced by SpCas9 can activate p53 pathway, which may induce cell cycle arrest and eventually apoptosis (Haapaniemi et al., 2018; Ihry et al., 2018). In order to avoid p53 activation and larger deletions, a CBE variant (A3A (N57Q)-BE3) with low off-target effect, has been used to disrupt the enhancer site with high efficiency (Zeng et al., 2020). Moreover, multiplex editing by disrupting *BCL11A* erythroid enhancer and repairing the common Chinese β-thalassemia promoter mutation *HBB* -28 A > G further enhanced γ-globin level (Zeng et al., 2020).

In addition to knocking out *BCL11A* erythroid enhancer, disrupting the *BCL11A* binding sites or other negative regulator binding sites on *HBG* promoter region is another way to alleviate the repression. CRISPR-Cas9 was used to generate a 13-nt deletion (−102 to −114) in the *HBG1/2* promoter, which could destroy the repressor binding motif and increase γ-globin expression (Metais et al., 2019; Traxler et al., 2016). However, large fragment deletion can be detected owing to high sequence similarity between *HBG1* and *HBG2* (Traxler et al., 2016). Noted that the editing efficiency by sgRNA targeting the HBG1 promoter significantly dropped down in the stem cell population, likely attributed to the ineffective microhomology repair pathway in HSC (Metais et al., 2019). Base editors such as CBE or ABE directly converts the base change without generating DSBs and the conversion is cell cycle independent. The void of DSB, particular at the complicated HBG locus, offers a better alternative. Using ABE targeting *HBG* promoter region, scientists have shown effective editing and elevated HbF in edited HSPCs with no detectable off-targets (Ling et al., 2020; Nicole Gaudelli, 2021). CBE, such as hA3A-BE3, could also reactivate γ-globin expression in HSPCs (Wang et al., 2020). However, the high activity of hA3A deaminase tends to generate sgRNA-independent editing (Jin et al., 2020) and overexpression of hA3A can induce DNA damage response and cell-cycle arrest (Landry et al., 2011). Therefore, there are still significant considerations to engineer a safer and controllable variant when transforming CBE into clinical practice.

Other than aforementioned regulatory sites, continued efforts have been spent to identify new motifs that regulate HBG expression, e.g. the −200 LRF-binding site (−197,−196 and −195) and the −158 region (−158,−152 and −151), which have been suggested to affect γ-globin expression (Weber et al., 2020), providing additional candidate sites for therapeutic treatment.

Apart from γ-globin reactivation, direct correction of sickle mutation is another way to treat SCD patient. In the initial efforts to correct sickle mutation in HSPC, scientists tried to use Cas9 to generate a DSB around the mutated site and delivery a repair template to guide the perfect repair, the latter of which can be delivered via integrase defective lentiviral vector (Hoban et al., 2016), adeno-associated viral vector 6 (AAV6) (Pattabhi et al., 2019; Romero et al., 2019), or chemical modified single-stranded DNA donor (Park et al., 2019; Pattabhi et al., 2019).Though these methods can achieve accurate gene modification with relative high efficiency in the bulk HSPC population, the efficiency dropped down at least by 70% in the hematopoietic stem cell (HSC) population as revealed by animal engraftment assays (Pattabhi et al., 2019). Clonal track of HDR-edited cells indicated substantial shrink of HSC clonal repertoire during hematopoiesis, raising safety concerns related to long-term repopulating activity of HSC (Ferrari et al., 2020). Nonetheless, several on-going phase 1/2 clinical trials using HDR strategy are being tested to treat SCD (NCT04205435, NCT04819841, NCT04774536).

Since HDR efficiency is relatively low in slowing dividing HSCs, direct restoration of sickle mutation via base editor offers a better choice. Indeed, using an engineered adenine base editor (ABE8e-NRCH) to convert the sickle allele (HBBS) into a non-pathogenic variant makassar allele (HBBG), Gregory A. Newby et al. showed up to 80% conversion of HBBS to HBBG and the editing efficiency persisted after animal engraftment experiments (Newby et al., 2021).

Apart from SpCas9 and its variants mediated editing in HSPCs, an engineered AsCas12a was employed for targeting *HBG1/2* promoter region in clinical trials to treat patients with SCD or β-thalassemia (Zhang et al., 2021, NCT04853576, NCT05444894). Although a number of clinical trials using CRISPR tools are being tested in hemoglobinopathies, more data and longer track of therapeutic outcome are still required to reach any further conclusions.

#### 4.1.2 Acute myeloid leukemia (AML)

AML is a hematological malignancy with abnormal proliferation of immature myeloid progenitors (De Kouchkovsky and Abdul-Hay, 2016; Vago and Gojo, 2020). The standard cares include chemotherapy and HSCT. In general, more than 60% patients have tumor relapse within three years post-HSCT and approximate 80% of them die within three years (Araki et al., 2016). Several new immunotherapies using chimeric antigen receptor T cells (CAR-T) or antibody drug conjugates (ADCs) targeting CD33, a well-established AML biomarker, have been tested in clinics or approved in the market (Bross et al., 2001; Castaigne et al., 2012; Jen et al., 2018; Marofi et al., 2021). Due to the presence of CD33 in normal myeloid progenitors, CD33-CAR T not only clear out tumor cells, but also wipe out myeloid progenitors, resulting in on-target off-tumor side effects, which explained the failure of CD33-CAR T clinical trials (Gill et al., 2014). CD33, a member of siglec family, is a negative regulator to allergy response (Crocker et al., 2007). *Cd33* knockout mouse has normal hematopoietic system without obvious defects (Brinkman-Van der Linden et al., 2003). Quite intriguingly, two teams have shown in humanized mouse model and non-human primate that knocking out *CD3*3 by CRISPR-Cas9 in donor HSPCs has little effect on hematopoietic reconstruction or gene expressions (Borot et al., 2019; Kim et al., 2018a). More importantly, the reconstituted *CD33*-knockout HSCT animal models, when challenged with tumor cells, are well tolerated with CD33 CAR-T or ADCs immunotherapy and have no side effects on normal myeloid lineage. This strategy is current being tested in Phase 1/2 clinical trials to evaluate the safety and efficacy (NCT04849910). This is another example of CRISPR-editing in facilitating CAR-T therapy by manipulating HSPCs to erase the side effect of targeting non-tumor cells, thereby attributing to a complete tumor specific biomarker that can be specifically recognized by CAR-T cells.

#### 4.1.3 Human immunodeficiency virus (HIV)

People infected with HIV have compromised immune system and require lifelong medication to keep the viral load at low level (Powell et al., 2016; Weiss, 1993). Chemokine receptor 5 (CCR5) is the major co-receptor mediating the entry of HIV-1 into immune cells (Deng et al., 1996). The naturally existed CCR5Δ32 allele, characterized by a 32-bp deletion in *CCR5* coding sequence resulting a premature truncated form of protein, is strongly associated with resistance to HIV-1 infection (Liu et al., 1996; Samson et al., 1996). Moreover, the “Berlin patient”, a HIV-positive acute myeloid leukemia patient, when treated with allogeneic HSCT from a donor harboring a homozygous *CCR5*Δ32 allele, fully recovered from AML and HIV infection (Allers et al., 2011). These observations have propelled the development of anti-HIV cell therapy by disrupting the virus-CCR5 interaction, particular in patient who are HIV-positive and suffered from leukemia. As proof of concept, *CCR5* knockout by CRISPR-Cas9 in HSPCs did not affect the reconstitution ability and cells become HIV resistant both *in vitro* and in mouse models (Xu et al., 2017). To further test the concept, *CCR5* ablated HSPCs was transplanted into a 27-year-old man with HIV-positive and acute lymphoblastic leukemia (NCT03164135; Xu et al., 2019). Nineteen-month post HSCT, the leukemia symptom in patient was in complete remission but HIV viral load was still high and patient required continued antiviral medication to control the viral load. The editing efficiency of *CCR5* disruption was approximately 5%. Considered that the Berlin patient received a homozygous *CCR5*Δ32 HSCT, that is 100% editing efficiency with one pure product, the failed HIV resistance is likely attributed to its low editing efficiency in the donor cells. Further pre-clinical investigations using base editors have indicated high disruption of *CCR5* ∼ up to 88% editing in HSPCs or T cells (Knipping et al., 2022). With the development of safer and more effective CRISPR tools, it is possible to cure HIV via CRISPR edited HSPCs in the near future.

#### 4.1.4 Primary immunodeficiency diseases (PIDs)

PIDs are a group of rare diseases that are caused by mutations in genes crucial in regulating immune cells development (Ten, 1998). Among them, severe combined immunodeficiency (SCID) is the most severe group in which infants are born without T or B cells and die within a year due to opportunistic infections (Shearer et al., 2014). Identifying a matched HSCT donor within such tight time window is very challenging and allogeneic transplantation are usually associated with severe GvHD. The idea of correcting mutated genes using autologous HSCT is very intriguing. Combing CRISPR-Cas system and AAV6 platform, Mara Pavel-Dinu integrated the full-length codon-optimized *IL2RG* cDNA into the endogenous *IL2RG* translational start site (Pavel-Dinu et al., 2019). Similarly, Rajeev Rai succeeded in making the site-specific integration of a *WAS* cDNA in the *WAS* genomic locus in HSPCs, which has the potential to cure Wiskott-Aldrich syndrome (Rai et al., 2020; Worth and Thrasher, 2015). Van Trung Chu led his team to repair gene mutation in the exon4 of *ELANE*, reaching up to 40% integration efficiency by HDR in severe congenital neutropenia (SCN)-derived HSPCs and restored neutrophil differentiation both *in vitro* and in humanized mouse models (Tran et al., 2020). It's also possible to repair the hot spot mutations, such as C676T in the exon7 of *CYBB* gene by co-delivery of SpCas9 mRNA, sgRNA and ssODN donor in X-linked chronic granulomatous disease patients derived HSPCs to restore nicotinamide adenine dinucleotide phosphate function (De Ravin et al., 2017). While these studies provide a proof-of-concept of using CRISPR-edited autologous HSPCs to treat PIDs, there are still several challenges to be solved prior to clinical applications. For example, PID are rare diseases with very small number of patients for each subtype. For each disease, the disease-causing genes are also different. In one specific gene-caused PID, the mutation spot varied patient to patient. Therefore, it is not practical to design a customized gene-editing product for each patient due to the complex drug development processes. Strategies such as integrating a mini-gene at a safe harbor locus or replacing the entire mutated gene could be a universal approach for each gene-caused disease. Continued efforts on engineering robust genome editing tools that allow gene insertion with high efficiency in non-cycling HSC will help develop new therapies for PID patients.

### 4.2 CRISPR in immune cells for immunotherapy

Immunotherapy is an emerging and promising therapy that modulate a person's immune system to treat cancer. Cancer immunotherapy is mainly divided into adoptive cell therapy (ACT) and immune checkpoint inhibitor (ICI) (Zhang and Zhang, 2020). ACT capitalizes on immune cells such as T cells, natural killer (NK) cells, macrophages to reactivate their antitumor response (Anderson et al., 2021; June et al., 2018; Xie et al., 2020). For example, immune cells can be engineered with chimeric antigen receptor (CAR) or recombinant T-cell receptor (TCR) that recognize tumor specific antigen, and have robust proliferation, normal exhaustion and minimal side effects. In addition to control the specificity of tumor recognition in T cells, downregulating immune checkpoint molecules in T cells or their ligands in tumors cells is another way to treat cancer (Bhatia and Kumar, 2014). For example, it's an attractive way to block immune checkpoints or their ligands such as knocking out programmed cell death 1 (PD-1) or cytotoxic T lymphocyte-associated antigen-4 (CLTA-4) (Pardoll, 2012) in engineered CAR-T or TCR-T cells. These would bring the immunotherapy to the next level, where CRISPR could certainly play an important role.

A first-in-human phase I clinical trial of CRISPR mediated *PD1*-knockdown in autologous T cells treating non-small-cell lung cancer was initiated in Sichuan university, China, which showed minimal adverse events and low off-target editing (Lu et al., 2020; Lu, 2016). Although the median editing efficiency was only 5.81% (range, 0.42–24.85%), it has preliminarily demonstrated the feasibility and safety of CRISPR editing T cells. In addition, a number of similar clinical trials targeting immune checkpoints including *PD1* and cytokine inducible SH2 containing protein (*CISH*) were conducted in patients with advanced esophageal cancer (NCT03081715), prostate cancer (NCT03525652), advanced stage Epstein-Barr virus (EBV) associated malignancies (NCT03044743), advanced Hepatocellular Carcinoma (NCT04417764), invasive bladder cancer (NCT02863913) and metastatic gastrointestinal cancers (NCT04426669). In terms of *CLTA-4,* some preclinical studies testified that knockout of *CTLA-4* can also strengthen anti-tumor activity in colon cancer and bladder cancer (Shi et al., 2017; Zhang et al., 2019).Though manipulating immune checkpoints in autologous T cells exhibited enhanced anti-tumor effect to some extent, it only triggered responses in selected patients.

CARs are genetic engineered receptors that enable tumor specific recognition without MHC-restriction (June et al., 2018; Sadelain et al., 2009). Although several autologous CAR-T cells targeting CD19 or BCMA have been approved by Food and Drug Administration (FDA) to treat relapsed or refractory large B-cell acute lymphoblastic leukemia, mantle cell lymphoma or myeloma, and CAR-T based ongoing clinical trials are in large numbers, there are still some limitations that need to be improved in order to further expand its applications. First of all, current CAR-Ts are generated via lentivirus or other viral vectors, which have safety concerns related to the viral vector mediated random integration into genome and heterogeneous CAR expression (Labbe et al., 2021; von Kalle et al., 2014; Wang and Riviere, 2016). The ability to achieve site specific insertions of CAR could help mitigate unstable genome toxicity and render a sustained and homogenous CAR expression for better efficacy (Zhang et al., 2022). Secondly, current CAR-T products are prepared from patients themselves, which can be an issue for infant patients or patients who do not have enough or high-quality T cells due to myeloablative therapies. The average CAR-T number required for infusion are in large quantity, around 1E8 per kilogram. The proliferative ability of patient T cells is highly variable, leading to uncontrollable cell manufactory (Levine et al., 2017). In some occasions, poor CAR-T responses are due to the intrinsic defect of patient T-cells (Fraietta et al., 2018). Thirdly, personalized CAR T-cells manufactory is not economical friendly and requires highly customized processes which could introduce more variations, affecting the final results. The idea of generating universal CAR-T cells from healthy donors could circumvent the issues associated with autologous T cells. In order to generate universal CAR-T cells, how to avoid GvHDs and immune rejection is the key for a successful allogenic infusion. The endogenous αβ T cell receptors (TCRs) from host cells can recognize mismatched human leukocyte antigen (HLA) from donor T cells, and the donor T cells can discern foreign HLA molecules from the host. Thus, silencing both TCR and HLA of allogeneic T-cells can protect donor T cells from recognizing host cells and help to escape the surveillance of host T cells (Glanville et al., 2017). One advantage of CRISPR system is its multiplex ability of generating simultaneous multi-gene knockout and gene knock-in. Justin Eyquem succeeded in combing electroporation of Cas9 mRNA and sgRNA with rAAV6 transduction carrying CAR cDNA to precisely insert CAR into the T-cell receptor α constant (*TRAC*) locus (Eyquem et al., 2017). They further showed that CAR-T generated via CRISPR outperformed than traditional retroviral-CAR T in both tumors killing ability and in T cell fitness. Further studies performed double knock-out (*TRAC, β2M*) or triple knock-out (*TRAC, β2M* and *PD1*) to generate the universal CD19-CAR T cells with high efficiency (Liu et al., 2017; Ren et al., 2017). These CRISPR edited UCAR-T cells exhibited no GvHD with anti-tumor activity both *in vitro* and *in vivo*. Moreover, triple knock-out CAR-T cells produced more interferon-γ with enhanced cell lytic activity than double knockout or standard CAR T-cells (Liu et al., 2017; Ren et al., 2017). This can be explained by the knock-out of T-cell suppressor *PD1* to alleviate the repression signaling (Pardoll, 2012). So far, there are 17 clinical trials based on CRISPR-generated allogenic CAR-T cell immunotherapy in early phase 1/2 (Table 1).

The other example that CRISPR could help with T-cell immunotherapy is to reduce the on-target off-tumor side effects. CD7 is a well-characterized T cell tumor marker which is also expressed in healthy donor T cells (Campana et al., 1991). The engineering of CD7 positive CAR-T could led to fratricide. To solve the problem, Diogo Gomes-Silva knocked out CD7 in donor T cells and then transduced CD7 CAR into edited cells, which not only restored normal proliferation but also eliminate the self-killing effect of CD7-CAR T (Gomes-Silva et al., 2017). Early phase I clinical trial has been started in patients with relapsed and/or refractory T-cell hematologic malignancies using CD7 CAR-T cells with *CD7* and *TRAC* knockout (NCT04264078). It's worth mentioning that a SpCas9-CBE editor is used in generating CAR-T cells, which is the first cytosine base editor that has been tested in human clinical trial (NCT05397184). Similarly, another clinical trial has initiated where *CD5* was disrupted in CAR-T cells for patients with relapsed/refractory CD5^+^ hematopoietic malignancies (NCT04767308).

### 4.3 CRISPR in induced pluripotent stem cells based cell therapy

Induced pluripotent stem cells (iPSCs) are a type of embryonic-like pluripotent stem cells that can be differentiated into various cell types such as neurons, hepatocytes, myocardial cells, and pancreatic β cells (Dimos et al., 2008; Lin et al., 2017; Yamanaka, 2012). iPSCs are derived from adult somatic cells by adding the classic Yamanaka factors to reprogram cell fate (Takahashi et al., 2007; Takahashi and Yamanaka, 2006; Yu et al., 2007). Due to the ease of iPSC production and its pluripotent ability, iPSC is a popular source for cell therapy. Unlike HSC, iPSC is actively dividing, which makes HDR based gene correction possible. Genetic mutations can be corrected *in vitro*, and consequently corrected iPSC derived cells can be reinfused back to patient.

The ability to differentiate iPSC into insulin-producing pancreatic β-like cells has opened a new path for treating diabetes. Diabetes mellitus is caused by dysfunction or death of insulin-producing β cells in the pancreas. Although routine insulin injections can help manage the disease manifestation, long-term complications can arise (Atkinson et al., 2014; Nathan, 1993; Schwartz et al., 2017). Allogeneic cadaveric islet transplantation is another way to cure diabetes (Meloche, 2007), demonstrating the feasibility of the cell replacement approach that is, however, limited by donor availability and the anti-rejection intervention (Shapiro et al., 2017). As a proof of concept, two research teams corrected the pathogenic mutation in *INS* or *WFS1* gene respectively in patient derived iPSCs by CRISPR-Cas9 gene editing tool (Ma et al., 2018; Maxwell et al., 2020). They were able to differentiate the edited cells into high insulin producing beta-like cells and proved its normal function in diabetic mice models (Maxwell et al., 2020). Another strategy of CRISPR tools in facilitating iPSCs based cell therapy is to engineer off-the-shelf iPSCs by manipulating the immune surveillance factors such as disrupting *β2M* gene and expressing a transgene encoding pro-tolerance programmed death-ligand 1 (*PD-L1*) simultaneously in order to suppress T cell activation (NCT05210530).

In addition to pancreatic beta cells, using iPSCs derived NK or T cells for immunotherapy is another actively investigated area. CRISPR-edited iPSCs can serve as universal NK or CAR-T donors used for treating blood and solid tumors. Unlike peripheral blood isolated NK cells, iPSC-NK cells derived from single clone, are homogeneous and have stronger *in vivo* response in mouse ovarian cancer and better performance than primary NK cells (Cichocki et al., 2020; Li et al., 2018; Liu et al., 2021b). When combined with CRISPR editing tools, iPSC-NK cells are significantly easier to obtain a homogenous edited population by selecting corrected edited cells in iPSC state and then differentiate into NK cells from a defined and reliable edited iPSC clone (Childs and Carlsten, 2015; Li et al., 2018). iPSC-derived off-the-shelf NK cells with *CD38* disruption has entered phase 1 clinical trial to treat acute myeloid leukemia, multiple myeloma, relapsed or refractory B-cell lymphoma and advanced solid tumors (Therapeutics, 2020; 2021a). CD38 disruption by CRISPR could increase antibody-dependent cellular cytotoxicity and enhance persistence. Several studies of iPSC-NK cell therapies with CRISPR genome editing to enhance innate immunity have been initiated for treating leukemia (Clara et al., 2022).

Though iPSC can be differentiated into various cell types, the relative low differentiation efficiency, the heterogeneous cell population, tumorigenicity and the ability to homing with *in vivo* environment are the challenges that need to be conquered prior to broad applications(Yamanaka, 2020). In the meantime, iPSC can be a powerful model for drug or sgRNA screening. For example, iPSC can be used to test different CRISPR editing strategies and sgRNA selection for duchenne muscular dystrophy (DMD). *DMD* gene is the largest gene in human genome ∼2.2 Mb, containing 79 exons (Koenig et al., 1987; Monaco et al., 1986). There are thousands of pathogenic mutations in *DMD* and direct correction of each mutation is not feasible. Common strategy involves the generation of exon skipping to restore the reading frame shift to rescue the normal function of muscle cells. The ability to differentiate into muscle cells and evaluate the muscle function has made iPSCs a great platform to screen sgRNAs that not only have high editing efficiency but also result in effective functional outcome (Kyrychenko et al., 2017; Min et al., 2019; Yuan et al., 2018; Zhang et al., 2017). Therefore, iPSC not only represent a great cell source for cell therapies, it is also a powerful sgRNA screening platform that propels the development of CRISPR-based therapies in many ways.

## 5 Conclusions and future perspectives

**Fig. 3:**
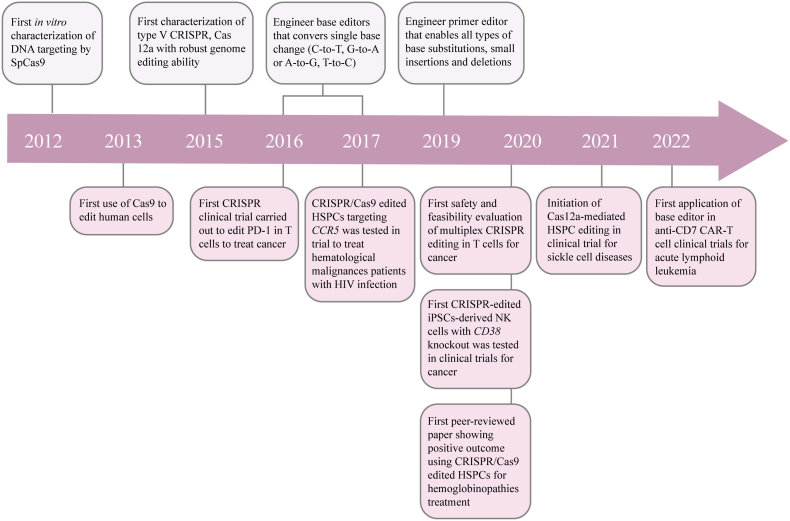
| **Major landmarks in CRISPR/Cas development and their progress in cell therapy.** Timeline highlighting the major developments in the fields of CRISPR-Cas based cell therapies, including the discovery of CRISPR toolbox and major clinical advances.

Over the past 10 years, we have witnessed the substantial development of CRISPR technology. Ever since its discovery, CRISPR has been quickly applied into mammalian genome modification and this has opened a new door to robustly manipulate human genome for basic research and translational therapy (Fig. 3). As of now, up to 300 CRISPR-Cas nucleases have been characterized and increasingly versatile and precise tools have been developed to fulfill the multiple aspects of biological research and of clinical application for disease treatment. These tools have revolutionarily established a foundation for the generation of new human therapeutic modality that can treat or eventually cure diseases.

CRISPR-based cell therapy has been at the forefront of this historical application due to its simplicity in delivery. The ability of generating variable editing types such as knockout, knock-in and base change with high efficiency, and the ability to combine multiplex editing in one reaction in difficult primary cells such as HSPCs and immune cells, has propelled CRISPR into many clinical trials for treating devastating diseases. Although these trials are still in their early stage, more and more trials are being green-lit and the preliminary data are very promising in support of the feasibility and efficacy. As we dive into more diseases that potentially can be treated with CRISPR, we are also aware there are still several challenges remain. 1) Gene writing in slow-cycling cells or non-cycling cells. The success could unlock full disease spectrum in HSC based therapies, such as PID and lysosomal storage disorders which previous have no cure but being limited to supportive care. The recently engineered twin PE paired with integrase holds great potential for large fragment deletion and insertion in slowing cycling cells. More work will be required to improve the safety and efficiency of this new system. 2) Improving target integration efficiency and safety in primary lymphocytes. The current strategy to delivery DNA donor template for target integration employs adeno-associated virus (AAV). Although effective, the manufacture process of AAV preparation is complicated and being the rate limiting step in CRISPR-based CAR-T therapy. In addition, the chance of viral backbone integration into host genome remains a safety concern. Direct electroporation of dsDNA into primary cells lead to extensive cell death (Gaidt et al., 2017; Murthy et al., 2020; Roth et al., 2018). Single-stranded ssDNA (ssDNA) can escape the surveillance of cGAS and enable effective delivery into nucleus (Shy et al., 2022), thereby serving as a promising delivery vehicle. Further studies to simplify the preparation of ssDNA or bypass dsDNA induced cell death will enable a safer and more effective target integration in lymphocytes. 3) Multiplex editing induced genome instability such as chromosomal translocation or the activation of p53 pathway induced risks of malignancy. 4) Bystander products and genome wide off-target effect. A recent structural analysis of Cas9 off-targets have suggested distal mismatches can be stabilized by a loop in RuvC domain (Bravo et al., 2022). It will be interesting to test whether mutating mismatch-stabilizing residues can reduce off-target effects without sacrificing on-target activity in cells. Other methods such as transient and controlled expression of genome editing enzyme could help alleviate off-target effects. 5) PAM restriction has restrained the broad application of base editors. Search for highly efficiency Cas nucleases with relaxed PAM is the key. Considering how much progress CRISPR technologies have been made since its discovery, we are hopeful that continued engineering and optimization over the next ten years will result a better system, unlocking new cell therapies to previous unsolved diseases and shedding lights on *in vivo* applications.

## Author contributions

HY.Q. and Y.Z. wrote the manuscript. RJ.J. drew the illustrations.

## Declaration of competing interest

The authors declare that they have no known competing financial interests or personal relationships that could have appeared to influence the work reported in this paper.
